# The Crucial Role of AR-V7 in Enzalutamide-Resistance of Castration-Resistant Prostate Cancer

**DOI:** 10.3390/cancers14194877

**Published:** 2022-10-05

**Authors:** Zeyuan Zheng, Jinxin Li, Yankuo Liu, Zhiyuan Shi, Zuodong Xuan, Kunao Yang, Chunlan Xu, Yang Bai, Meiling Fu, Qiaohong Xiao, Huimin Sun, Chen Shao

**Affiliations:** 1 Department of Urology, Xiang’an Hospital of Xiamen University, School of Medicine, Xiamen University, Xiamen 361101, China; 2 Department of Nephrology, Xiang’an Hospital of Xiamen University, School of Medicine, Xiamen University, Xiamen 361101, China; 3 Central Laboratory, Xiang’an Hospital of Xiamen University, School of Medicine, Xiamen University, Xiamen 361101, China

**Keywords:** AR-V7, enzalutamide, drug resistance, castration-resistant prostate cancer, proteostasis, LncRNA

## Abstract

**Simple Summary:**

Androgen receptor splice variant 7 (AR-V7) has always been considered a key driver for triggering enzalutamide resistance of castration-resistant prostate cancer (CRPC). In recent years, both the homeostasis of AR-V7 protein and AR-V7’s relationship with LncRNAs have gained great attention with in-depth studies. Starting from protein stability and LncRNA, the paper discusses and summarizes the mechanisms and drugs that affect the CRPC patients’ sensitivity to enzalutamide by regulating the protein or transcriptional stability of AR-V7, hoping to provide therapeutic ideas for subsequent research to break through the CRPC therapeutic bottleneck.

**Abstract:**

Prostate cancer (PCa) has the second highest incidence of malignancies occurring in men worldwide. The first-line therapy of PCa is androgen deprivation therapy (ADT). Nonetheless, most patients progress to castration-resistant prostate cancer (CRPC) after being treated by ADT. As a second-generation androgen receptor (AR) antagonist, enzalutamide (ENZ) is the current mainstay of new endocrine therapies for CRPC in clinical use. However, almost all patients develop resistance during AR antagonist therapy due to various mechanisms. At present, ENZ resistance (ENZR) has become challenging in the clinical treatment of CRPC. AR splice variant 7 (AR-V7) refers to a ligand-independent and constitutively active variant of the AR and is considered a key driver of ENZR in CRPC. In this review, we summarize the mechanisms and biological behaviors of AR-V7 in ENZR of CRPC to contribute novel insights for CRPC therapy.

## 1. Introduction

As the most common urological malignant tumor, prostate cancer (PCa) occurs in the prostate epithelium, and is the fifth leading cause of death from cancer in men. New cancer cases among men worldwide exceeded 10 million in 2020, 1.4 million of which were newly diagnosed with PCa and accounted for 14.1% of all incident cases in men. And the number of deaths from PCa was 375,304, making up 6.8% of all cancer deaths in men [1]. The incidence of PCa, ranking the highest in 112 countries and regions, is lower in East Asia than that in European and American countries, whereas men in East Asia had higher PCa mortality rates than those in European and American countries [2]. The occurrence and development of PCa are closely related to androgens that can stimulate the growth of PCa cells and promote the progression of PCa. Consequently, PCa is an androgen-dependent tumor [3]. For this reason, androgen deprivation therapy (ADT) has become the standard treatment for PCa. It slows the progression of PCa by reducing androgen levels in the body or inhibiting the androgen receptor (AR) through surgery or medical castration such as the application of AR signaling inhibitors (ARSI). However, most patients develop drug resistance within 2 to 3 years after ADT and progress to castration-resistant prostate cancer (CRPC) [4,5].

The introduction of second-generation ARSI has greatly impacted the treatment of CRPC [6]. Enzalutamide (ENZ) is the second-generation ARSI by the FDA approval and the most important new drug of endocrine therapies for CRPC [7]. ENZ is one of the important options for both metastasis CRPC (mCRPC) and non-metastatic CRPC (nmCRPC) patients [3]. A phase III clinical trial investigating the efficacy and safety of ENZ in patients with nmCRPC showed that nmCRPC patients treated with ENZ had significantly improved metastasis-free survival, suggesting that ENZ significantly improves overall survival in nmCRPC [8]. Additionally, a recent meta-analysis also supported this view [9]. More than this, the overall survival and progression free survival of mCRPC patients treated with ENZ were prolonged, and the five-year survival rate was significantly improved [10,11].

ENZ mainly acts through the blockade of the AR signaling pathway, including blocking the binding of androgens to AR, impeding the AR nuclear translocation, and inhibiting the AR transcriptional activity for the treatment of CRPC [4,12,13]. Scher et al. demonstrated through a double-blind placebo-controlled trial the median survival of 18.4 months of the ENZ-treated patients, compared with 13.6 months in the placebo group [14]. ENZ thereby can significantly improve the survival of patients with CRPC after chemotherapy. However, Azuma et al. showed the insensitivity of some patients with CRPC ENZ during the clinical application of ENZ [15]. Also, drug resistance still occurred even after 11.2 years since the effective primary treatment [16]. These findings indicate that drug resistance develops through multiple mechanisms in nearly all patients with PCa treated by ARSI.

AR-related signaling pathways have always been seen as one of the considerable mechanisms responsible for ENZ resistance (ENZR) in CRPC, such as the aberrant amplification and/or overexpression of AR [17,18], AR mutation [19,20], generation of androgen splice variants (AR-Vs) [20]. Further, ENZR in CRPC has been strongly affected by neuroendocrine prostate cancer (NEPC) as a result of AR-mediated lineage plasticity [21], and by the activation of related signaling pathways like Wnt, PI3K, and NF-kB [22,23]. The insensitivity of CRPC to ENZ has been attributed in a large part to the generation of AR-V7 [24,25]. A plethora of research in this field centered on the relationship between cofactors and AR-V7. The transcriptional coactivator four-and-a-half LIM domain protein 2 (FHL-2) can act as an AR-V7 agonist [26]. Vav guanine nucleotide exchange factor 3 (VAV3) can promote the activity of AR-V7 to enhance the progression of CRPC [27,28]. Moreover, cofactors can affect ENZR through the interaction with AR-V7, such as mediator complex subunit 1(MED1) [29], fork head box01 (FOX01) [30], GATA binding protein 2 (GATA2) [31], and src-associated in mitosis of 68KD (SAM68) [32]. Nowadays, related research has paid attention to proteostasis and the role of Long noncoding RNA (LncRNA) in cancer. The changes of AR-V’s protein homeostasis are closely related to prostate cancer, and its relationship with LncRNA has also attracted the attention of researchers and has been studied in depth. This article reviews and summarizes the related research, to provide clues to solve ENZR and to improve the treatment of CRPC.

## 2. The Relationship between AR-V7 and AR-FL

### 2.1. AR-V7 Is Derived from the Splicing of AR-FL

The full-length AR (AR-FL) is mapped to chromosome Xq11-Xq12 [33]. For the first time, Chang et al. cloned the AR cDNA of human beings and rats in 1988 [34]. AR-FL is approximately 919 amino acids [35,36], mainly consisting of four structures which are the N-terminal domain (NTD), the DNA-binding domain (DBD), the hinge domain (HD), and the ligand-binding domain (LBD) [37]. AR-Vs result from alternative splicing of AR-FL during the treatment of CRPC using the second-generation ARSI. Currently, the mechanism that underlies the production of AR-Vs has been mostly understood by AR gene rearrangements [38,39], which is the primary mechanism leading to the generation of AR-V7 and AR-V567es [40], point mutations in the AR gene [41], post-translational modifications [42], and involvement of related splicing factors such as heterogeneous nuclear ribonucleoprotein A1 (hnRNPA1) [43], U2 small nuclear RNA auxiliary factor 2 (U2AF2) [44], Sam68 [45].

### 2.2. AR-V7 and AR-FL Have Similar Structures

**Figure 1:**
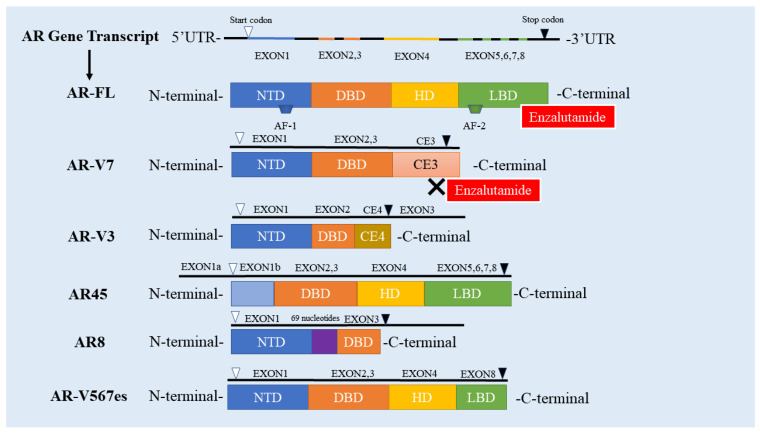
The Structure of AR-FL and AR-Vs. The human AR gene contains eight exons; AR has four domains, including NTD, DBD, HD and LBD. Exon 1 encodes NTD, DBD is encoded by exons 2 and 3, exon 4 encodes HD, and LBD is encoded by exons 5,6,78; AR-V7 has no LBD, so enzalutamide cannot bind to AR-V7; The DBD domain of AR-V3 is only encoded by exon 2, and lacks zinc finger; AR45 starts coding from exon 1b and lacks the NTD domain; The DBD domain formed by AR8 is only encoded by exon 3 and is not functional. AR8 inserts 69 nucleotides upstream of exon 3; AR-V567es does not contain exon 5.6.7; UTR: Untranslated Region; AR-FL: full-length androgen receptor; AR-V: androgen receptor splicing variant; NTD: N-end domain; DBD: DNA binding; HD: hinge domain; LBD: ligand binding domain; CE3: recessive exon 3; AF: Activate the function.

**Figure 2:**
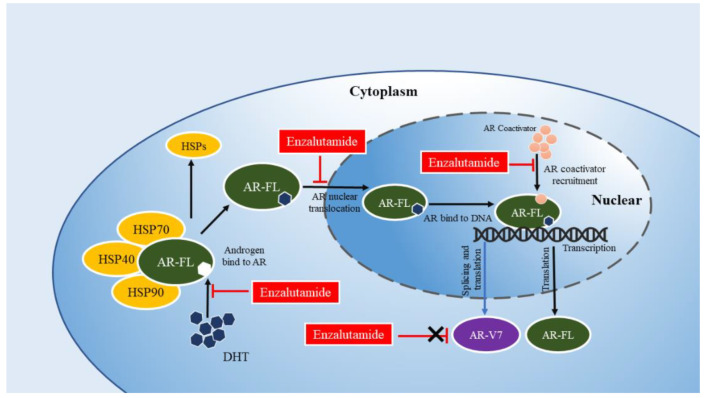
Enzalutamide acts on the AR signaling pathway. Enzalutamide binds to the LBD and blockades the AR signaling pathway by blocking the binding of androgens to AR, impeding the AR nuclear translocation, and inhibiting the AR transcriptional activity. AR-FL: full-length androgen receptor; AR-V7: androgen receptor splice variant 7; DHT: dihydrotestosterone. HSP: heat shock proteins.

AR-Vs were first discovered over 20 years ago and reported by Tepper et al. [46]. At present, more than 20 types of AR-Vs have been found and identified [47], the majority of which contain complete DBD and NTD (AR8 with the lack of the functional DBD and AR-V3 with no zinc fingers of DBD, with the exception of AR45 that does not contain NTD). Most of them have primarily LBD and HD loss [47]. AR-V7 retains the complete NTD and DBD while lacking the LBD and HD which is displaced by a short peptide sequence encoded by cryptic exon 3 (CE3) instead [48]. Therefore, AR-V7 that contains a complete activation functions (AF)-1 transactivation domain is constitutively active at the transcriptional level and maintains gene transcription without the need for ligand (independent of androgen) binding [49]. Given that ENZ targets LBD [50], the truncated LBD in AR-V7 is the primary cause why ENZ cannot bind to the AR-V7 and further play its role, subsequently leading to ENZR (Figure 1 and Figure 2).

### 2.3. There Are Differences between AR-V7 and AR-FL

AR-V7 is a mimic of AR, or AR-V7 has unique activity or whether AR-V7 contains a unique binding site is the focus of researchers. A recent study has found that AR-V7 induces a slightly higher proportion of total regulatory genes in the cell model than AR-FL, and its differential binding with chromatin is stronger than AR-FL [51]. Some studies also have shown that AR-V7 has specific targeted genes, which cannot be targeted by AR-FL [52]. These results together indicate that AR-V7 has unique activity and is not just a mimic of AR-FL.

### 2.4. AR-V7 Plays a Role Independently of AR-FL

AR-V7 is derived from AR-FL and is almost always co-expressed with AR-FL in the clinical environment. Therefore, the topic of whether the role of AR-V7 in PCa depends on AR-FL has been widely debated. Some researchers have concluded that AR-V7 works with AR-FL or requires AR-FL to perform activities, because AR-V transcriptional activity requires AR-V homologous dimerization [53]. AR-V7 can also form heterodimers with AR-FL [54]. In addition, in the model of the expression of AR-FL, AR-V7 activity depends on AR-FL to a large extent [55,56]. However, other researchers believe that exogenous overexpressed AR-V7 can act as a heterodimer of AR-FL, but whether the endogenous AR-V7 functions depend on heterodimerization with AR-FL remains to be further studied, because in AR-FL null cell models and/or AR-FL depleted models, many AR-Vs have been proven to have the ability to regulate gene transcription without AR-FL [48,57,58]. Similarly, the experimental study of Liang et al. also emphasized that AR-FL deletion would not damage the chromatin binding of ARv7 splicing variants, and that AR-FL and AR-V7 independently participated in AR transcriptional activity [59], which seems to be a shred of strong evidence that AR-V7 plays a role independently of AR-FL.

## 3. The Close Relationship between AR-V7 and the Treatment of CRPC with ENZ

Sharp et al. assessed the expression of AR-V7 protein in 358 primary prostate specimens and 293 metastatic biopsy specimens [60]. The results showed a very low level (<1%) of the expression of AR-V7 protein in cases with primary PCa and a detectable level of in 75% in cases treated by ADT. A marked increase in the expression of AR-V7 protein in cases with ENZ demonstrated that the use of ENZ can enhance the generation of AR-V7. However, the use of ENZ is not an absolute factor in the production of AR-V7, because AR-V7 expression is also detected in patients with primary PCa. This is also consistent with the research of Sciarra et al. [61]. They classified 56 cases of PCa according to clinical risk, examined the expression of AR-V7 and found that 24 of 32 high-risk patients were AR-V7 positive, 4 of 13 in the medium risk group and 2 of 11 in the low risk group. Therefore, AR-V7 could be detected by immunohistochemistry in more than 50% of nmPCa patients without treatment. It also showed that there was a significant correlation between AR-V7 positive and PCa risk classification (*p* < 0.001)

The generation of AR-V7 is one of the causes of the poor prognosis and low overall survival rate of CRPC patients after treatment. Compared with AR-V7 negative patients, the prognosis of PCa patients treated with ENZ became worse if they were also AR-V7 positive [62]. The research team selected 31 men using ENZ and Abiraterone (ABI) respectively. Among the patients treated with ENZ, it was found that the PSA response rate of AR-V7 positive ones was lower than that of negative ones (0% vs. 53%, *p* = 0.004). Moreover, it was also found that the AR-V7 positive patients had a shorter PSA progression-free survival (median 1.4 months vs. 6.0 months; *p* < 0.001), shorter clinical or radiographic progression-free survival (median 2.1 months vs. 6.1 months; *p* < 0.001) and shorter overall survival (median 5.5 months vs. not reached; *p* = 0.002), indicating insufficient therapeutic efficacy and prognosis as a consequence of the decreased sensitivity of AR-V7 positive patients to ENZ. Similarly, research detected in various ways the AR-V7 in the CTCs of blood samples belonging to high-risk CRPC patients being treated by ENZ and came to the same conclusion: the AR-V7 expression is independently related to shorter clinical progression-free survival (PFS) and poorer overall survival (OS) [63]. Meanwhile, it further showed that the continuous subsequent treatment with ENZ would not result in a good endpoint for patients with CRPC who were AR-V7 positive. Therefore, ENZ leads to the generation of AR-V7 and drug resistance, which impedes the therapeutic efficacy and survival rate of patients. Up to now, the treatment of AR-V7 positive CRPC patients has become a challenging issue in the clinic.

A large number of preclinical research results show that AR-V7 can be used as a biological marker of ENZR in CRPC [64,65]. Besides, AR-V7 is considered helpful in guiding the treatment selection after ABI or ENZ progress in mCRPC. It has available commercial test methods and has been prospectively validated in multiple centers [66]. Therefore, improving the detection efficiency and accuracy of AR-V7 can help not only patient diagnosis but also the precise guidance of medical use for CRPC patients in the clinic. As previously mentioned, Zhu et al. developed an analytical method of in situ hybridization with AR-V7 RNA. The technology can overcome the limitation that AR-V7 cannot be accurately detected by observing single splicing connections in cells and tissues [67]. Maillet et al. applied enrichment analysis (the AdnaTest) to detect the mRNA-based AR-V7 in circulating tumor cells (CTCs), and performed the pre-amplification PCR step before real-time qPCR analysis to establish a highly sensitive method for detecting in CTCs AR-V7, and significantly improves the detection efficiency of AR-V7 [68]. Despite that the clinical utility of these detection methods remains controversial, they do offer new hope to patients with CRPC who are ENZR to get early treatment.

## 4. AR-V7 as the Cause of ENZR in CRPC

AR-V7 has long been a crucial research focus in exploring the mechanism of ENZR. In cell-level experiments, it has been found that the ENZ therapy of CRPC enhances the level of AR-V7 and aids the expression of its target genes in PCa cells, and that the knockdown of AR-V7 can restore the sensitivity of cancer cells to ENZ, suggesting that AR-V7 is one of the salient factors leading to drug resistance [69]. Similarly, Zhu et al. found that patient-derived xenografts (PDX) with high expression of AR-FL could resist the first-generation ARSI but not the second-generation ARSI ENZ, proving that ENZR was closely related to AR-V7 expression [70]. The authors further demonstrated through subsequent cell experiments that ENZR required the high-level AR-FL and critical-level AR-V7 expression. Although the precise mechanism by which AR-V7 plays its role remains unknown, all the above indicates that AR-V7 is crucial in mediating ENZR of CRPC. Moreover, basic research has centered on the change of AR-V7 proteostasis and how its interaction with LncRNAs works on ENZR in CRPC in recent years, which also provides new therapeutic insights for the treatment of drug resistance in CRPC.

### 4.1. Heat Shock Protein Participates in Regulating the Protein Stability of AR-V7

Heat shock proteins (HSP) refer to a group of proteins generated by cells under the induction of stressors, especially environmental high temperature [71]. They are highly conservative molecular chaperones, including HSP70, HSP40, HSP90, etc. [72,73,74,75,76]. As molecular chaperones, HSPs regulate the stability and function of client proteins in a variety of ways, including preventing the aggregation of misfolded proteins, promoting intracellular protein transport, maintaining protein conformation to achieve ligand binding, and promoting the degradation of severely damaged proteins by mediating the ubiquitin proteasome [77,78].

**Figure 3:**
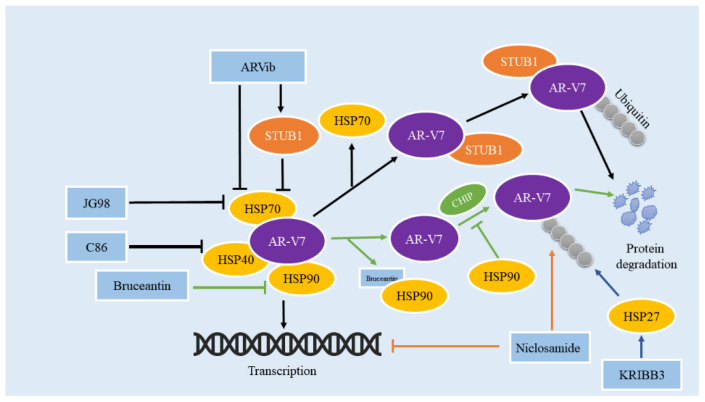
Natural/compound drugs affect the transcriptional activity and protein stability of AR-V7 through the HSP family. ARVib, JG98, and C86 promote AR-V7 degradation by inhibiting HSP40/HSP70 axis; bruceantin promotes AR-V7 degradation by directly binding to HSP90 and disrupting the interaction between HSP90 and AR-V7; KRIBB3 promotes the degradation of AR by interacting with HSP27; niclosamide not only inhibits the transcriptional activity of AR-V7 but also promotes the degradation of AR-V7.

AR/AR-V7 is one of the client proteins of HSP [79]. In the cytoplasm, free AR/AR-V7 combines with chaperone HSP70-HSP40 to form a protein complex, and then HSP90 and chaperone bind to AR/AR-V7, which dissociates AR/AR-V7 from HSP70-HSP40. HSP90 can ensure that AR/AR-V7 retains the conformational structure with a high affinity for its ligand dihydrotestosterone, and promote the binding of AR/AR-V7 with ligand [80]. After AR binds dihydrotestosterone, phosphorylation and dimerization occur. The combination of HSP27 and AR homodimer further promotes the nuclear translocation of the androgen response element (ARE) in AR and specific gene promoter regions, thus promoting the regulation of transcriptional activity [81]. A promising strategy for the treatment of cancer is to increase the degradation of oncogenic proteins by targeting the ubiquitin-proteasome system [52,53]. Therefore, it is one of the potential strategies to affect the protein stability of AR/AR-V7 or treat the resistance of CRPC to ENZ by regulating heat shock proteins (Figure 3).

HSP70 has been identified as a biomarker for the survival and prognosis of PCa owing to the relationships between its overexpression and carcinogenesis as well as drug resistance in PCa [82]. HSP70 inhibitor JG98 can impede protein levels and transcriptional activities of AR-FL and AR-Vs, and degrade other oncogenic proteins at the same time [77,83]. Moses et al. also revealed that PCa patients who had developed drug resistance to the standard antiandrogen therapy would benefit from targeting the Hsp40/Hsp70 chaperone axis [83]. One of the reasons that niclosamide, an anti-worm drug, has been proven to be an effective inhibitor to reverse ENZR in CRPC, is that it can promote the protein degradation of AR-V7 by activating the ubiquitin protease hydrolysis pathway [84]. STIP1 homology and U-box containing protein 1(STUB1) is a co-chaperone protein and a functional E3 ubiquitin ligase [85]. AR/AR-V7 proteostasis requires the interaction of STUB1, the E3 ubiquitin ligase, and the HSP70 complex. STUB1 separates AR/AR-V7 from HSP70, provoking the ubiquitination and degradation of AR/AR-V7 [86]. Liu et al. found an analog that targeted and degraded AR/AR-V7 through a niclosamide analog library. It was identified and termed ARVib [87]. ARVib-7 which had a higher bioavailability than niclosamide, can inhibit the proliferation of AR-V7 transcriptional activity in PCa cells. Follow-up experiments discovered that ARVib-7 can inhibit the AR-V7 transcriptional activity in cells of ENZR in PCa, and also that it was able to promote the nuclear translocation of STUB1 by inhibiting the expression of HSP70. To treat drug resistance in CRPC, the increased nuclear penetration of STUB1 further binds to AR-V7 to facilitate its ubiquitination and degradation and to impair the expression of AR-V7 downstream genes in PCa cells. All eukaryotic cells contain the expression of the stress protein Hsp90, which is essential for the correct folding, hormone binding, and transcriptional activity of AR.

Moon et al. screened and identified the drug bruceantin (BCT) from a small antimalarial drug library, by directly binding to HSP90 which can not only disrupt the interaction between HSP90 and AR-FL/AR-V7 but also inhibit the chaperone function of HSP90, causing the degradation of AR-FL/AR-V7 through the ubiquitin-proteasome system [88]. These results indicate that BCT is a promising option for anti-CRPC medication. Geldanamycin (GA), the Hsp90 inhibitor, has also been reported to impair the AR proteostasis with no impact on the protein level of AR-V7. In the same way, it can impede AR transcriptional activity but has little impact on AR-V7 activity. All uncovered that AR-V7 is resistant to Hsp90 inhibitors [89]. Similar results have been reported in an article, and it is important to note that the second-generation HSP90 inhibitor Onalespib mainly affects the splicing of AR-V7 rather than directly affecting the protein levels of AR-V7 [90]. This experiment showed that Onalespib was able to induce AR-FL proteasomal degradation but had little effect on AR-V7 stability, and it was able to cause a decrease in AR-V7 mRNA levels, but did not affect total AR transcript levels [91]. Clearly, a close connection exists between HSPs and AR/AR-V7. Further research is required to confirm the impact of the HSP90 inhibitor on the proteostasis of AR-V7 and to investigate the role of other HSPs.

### 4.2. Natural Products and Compounds Can Regulate the Stability of AR-V7

In recent years, the application of natural products and their analogues in tumors has received much attention. AR-V7 is a substrate for the proteasome, as Liu et al. demonstrated [92]. They extracted nobiletin from citrus peel, a type of polymethoxylated flavonoid, which induced cancer cells to block in the G0/G1 phase and prevented the interaction of AR-V7 with ubiquitin specific peptidase 14 (USP14) and USP22, so as to selectively stimulate the proteasome destruction of AR-V7. AR-V7 positive PCa cells were thereby more sensitive to ENZ. In addition, this study highlighted that nobiletin was able to downregulate AR-V7 in a manner dependent on dose and time, but had no significant effect on the protein level of AR-FL. Previous research has also reported that nobiletin inhibits the growth of PCa cells by impeding the signaling pathway of TLR4/TRIF/IRF3, TLR9/IRF7, and AKT [93,94]. Huaier extract, derived from medicinal fungi, has been found to be anti-proliferative in CRPC cells. By inhibiting the transcriptional activity and nuclear translocation of AR-FL/AR-V7, it enhances the proteasome-mediated degradation of AR-FL/AR-V7 proteins through the down-regulation of USP14 and can consequently be applied as an effective inhibitor of AR-FL and AR-V7 in PCa [95]. Coincidentally, Liao et al. screened and identified rutin (Rut), the main component of Evodia rutaecarpa, from the natural product library, and revealed that it can be used as an inhibitor of AR-V7 [96]. They demonstrated that this alkaloid inhibited AR-V7 signaling by selectively accelerating the degradation of the K48-binding ubiquitinated proteasome.

A novel protac small molecule named MTX-23 was designed and developed by Lee et al. [97], which was made to bind both the VHL ligand of E3 ubiquitin ligase and the DNA binding domain (DBD) of AR simultaneously. It was found in the research that MTX-23 had no crucial effect on the mRNA level of AR-FL and AR-V7, and that, however, MTX-23 can specifically stimulate the ubiquitination and degradation of AR-V7 and AR-FL through the VHL E3 ligase/proteasome axis at the protein level to inhibit drug resistance. Interestingly, the authors discovered that MTX-23 was more effective than AR-FL in the degradation of AR-V7 (DC 50 of 0.37 and 2μmol/L respectively), given the fact that MTX-23 played its role through targeting DBD substantially. Theoretically speaking, MTX-23 should be able to degrade both AR-V7 and AR-FL with similar efficacy. The LBD remaining in AR-FL or the protein bound to LBD, according to the authors, may physically interfere with the interaction of MTX-23 with DBD, as a consequence of the possibility that the biological outcomes of MTX-23-mediated ubiquitination may vary in AR-V7 and AR-FL.

One approach to treating cancer is to screen and create small molecule compounds from databases. Nevertheless, continuous discovery of natural products and their derivatives has demonstrated an extraordinary anti-cancer effect as well as their abilities to reverse tumor resistance, suggesting the crucial clinical value of natural products and their derivatives (Table 1). Moreover, there are still many possibilities for the development in the utilization of natural products and their derivatives to treat ENZR in CRPC. Deep research will develop stronger and safer medications against drug resistance in the treatment of tumors.

### 4.3. LncRNA Has Multiple Effects on AR-V7

LncRNA has been widely researched in the past decade, which is defined as an RNA transcript longer than 200 nucleotides without protein coding [110]. Earlier research has shown that LncRNAs play an important role in cell cycle regulation [111], cell differentiation regulation [112], cell metabolism regulation [113], and other cellular processes. Meanwhile, LncRNAs can regulate its transcriptional level and epigenetic modification through interaction with DNA, proteostasis, and so on [114,115].

Recent research has displayed close relationships between LncRNAs and the occurrence and development of malignancies [116,117]. LncRNA-PCA3 has evolved into the most PCa-specific LncRNA which is an important diagnostic marker of PCa [118,119,120]. LncRNA-HOTAIR and LncRNA-GAS5 also play an indispensable role through various mechanisms in the progression of CRPC [121,122]. Shang et al. found that LncRNA-PCAT1 regulated PHLPP/FKBP51/IKK α complex to activate AKT and NF-κB signaling [123]; Wen et al. identified and termed a special LncRNA that transcribed at the CHPT1 enhancer as CHPT1-eRNA, and discovered that the level of the LncRNA in ENZR tumors was higher than that in the control group. Further cell experiments showed that this LncRNA was engaged in the generation of SE and regulated the transcriptional activity of SE by directly binding to BRD4, thus significantly provoking ENZR in CRPC [124]. Together, all research mentioned above revealed the status of LncRNAs in CRPC.

Wang et al. revealed the relevance between LncRNAs and the expression of AR-V7 in CRPC. Specifically, LncRNA-Malat1 is essential for the generation of AR-V7 [125]. They focused on the role of 32 LncRNAs associated with drug resistance in CRPC and verified how they worked in ENZR in CRPC through the construction of multiple ENZR-PCa cells and human clinical data. The authors mainly discussed the role of LncRNA-PCGEM1 and LncRNA-Malat1 in the mechanism of drug resistance, with LncRNA-PCGEM1 being identified as an AR/AR-V7 signal-regulated LncRNA [126], and LncRNA-MALAT1 proved to be upregulated in CRPC and to be likely to participate in RNA splicing [127,128]. They also discovered the correlation between the rise in SF2 activity and the overexpression of LncRNA-MALAT1 in ENZR C4-2 cells. Their previous research pointed out that introns between exon 3 and exon 4 of AR transcripts can be recognized and bound by SF2, which aided the generation of AR-V7 and consequently led to ENZR [129]. Meanwhile, knockdown of LncRNA-Malat1 with siRNA to inhibit the expression of LncRNA-MALAT1 leads to a significant reduction in AR-V7 expression, indicating that LncRNA-Malat1 is indispensable for ENZ induced AR-V7 expression.

The LncRNA was related to the generation of AR-V7. Additionally, the most recent research uncovered that LncRNA might also change the expression level and/or function of AR-V7 through a series of mechanisms, affecting the drug sensitivity of ENZ in CRPC. Zhang et al. found that LncRNA-PCBP1-AS1 can accelerate the progression of ENZR in CRPC by binding to NTD to improve the stability of the USP14-AR-V7 complex. AR-V7 and USP22 were found in this experiment to be greatly reduced by PCBP1-AS1 depletion [130]. Therefore, targeting PCBP1-AS1 could impede the expression of AR-V7 and strengthen the sensitivity of CRPC to ENZ. In 2022, Ghildiyal et al. discovered a type of LncRNA named NXTAR with the ability to inhibit drug resistance in PCa, and reported that NXTAR directly bound to AR upstream of the AR promoter to enhance EZH2 recruitment, leading to the down-regulation of the AR and AR-V7 expression and positive feedback to increase NXTAR expression [131]. This discovery provides a therapeutic strategy for treating recurrent CRPC. As previously mentioned, the role of LncRNAs in ENZR in CRPC has been further expanded, which may serve as new potential for clinical diagnosis and targeted therapy.

NTD is one of the necessary structures for AR transcription. Hirayama et al. [54] found that antagonizing NTD can decrease the transcriptional efficacy of AR-FL and AR-V7, effectively improving the efficacy of ENZ [69]. Targeting AR-NTD is thus one of the effective ways to inhibit AR-V7 and another option to treat ENZR in CRPC. Zhang et al. reported that LncRNA-KDM4A-AS1 enhanced the stability of the USP14-AR/AR-Vs complex and promoted the ubiquitination of AR/AR-Vs through the binding with NTD to protect itself from the MDM2-mediated degradation of ubiquitin-proteasome, thereby intensifying ENZR in CRPC. At present, many medicines designed to target AR-V7 NTD are under development [132]. This also suggests that targeting other structures in AR-V7 may have the same effect, such as DBD.

Generally speaking, LncRNAs and AR-V7 are closely related to each other, playing an important role through various mechanisms in ENZR in CRPC (Table 2). In addition, LncRNA-P21 [133], LncRNA-H19 [134], and so forth have also been reported to promote the occurrence of NEPC and consequently lead to ENZR. Therefore, targeting LncRNA and AR-V7-related signaling pathways may contribute new insights for the clinical treatment of drug resistance in CRPC.

### 4.4. Interaction between Other Molecules and AR-V7

Recent studies have reported that many molecules regulate the expression of AR-V7 in different ways (Table 3). AKR1C3, a member of the NAD(P)H-linked oxidoreductase superfamily, is highly expressed in PCa and its precancerous tissues in the human body [139]. Previous research has demonstrated that AKR1C3 can provoke ENZR by triggering the androgen biosynthesis pathway and AR signaling [140]. Through the ubiquitin-mediated proteasome pathway, AKR1C3 can induce the generation of AR-V7 and enhance its proteostasis. Indomethacin, an AKR1C3 inhibitor, can be utilized through the ubiquitin-mediated proteasome pathway targeting AKR1C3, to significantly reduce the expression of AR/AR-V7 protein in vitro and in vivo, and thus the sensitivity of patients to ENZ increases [101]. This is consistent with the findings of Wang et al. who found that AKR1C3 bound to AR-V7 protein in CRPC cells and mutually improved the proteostasis of AR-V7 and AKR1C3 by inhibiting ubiquitin activity in a positive feedback mechanism [102].

The kinesin family encompasses kinesin family member 15 (KIF15) which is essential for the development of bipolar spindles [141]. KIF15 is directly bound to the N-terminal of AR/AR-V7 to increase the binding between USP14 and AR/AR-V7 protein, thereby inhibiting the degradation of AR/AR-V7 protein and promoting ENZR [142]. Deleted in breast cancer 1 (DBC1) is a human nuclear protein that modulates the activities of various proteins [143]. Su et al. discovered that DBC1 interacted with AR-V7 to enhance the DNA binding activity of AR-V7 and competed with CHIP for AR-V7 binding to inhibit the ubiquitination and degradation of AR-V7 mediated by the CHIP E3 ligase, and thus stabilizing and activating AR-V7 [144]. As a result, the possibility exists in this situation that DBC1 could be targeted to decrease the stability of AR-V7 or reverse ENZR in CRPC. Aberrant expression of casein kinase 2 (CK2) is associated with tumorigenesis and progression of PCa [145], the novel CK2-selective inhibitor CX4945, which selectively downregulates AR-V7 at the mRNA and protein levels, offers a potential option for the treatment of PCa, especially CRPC [103].

How AR-V7 mediates genome function in CRPC remains largely unknown. homeobox B13 (HOXB13), a member of the homeobox proteins family, is a key regulator of the epithelial differentiation in the prostate gland [146]. Zhong et al. reported that HoxB13 firstly combined with AR-V7 through direct physical interaction, and cooperated with AR-V7 to up regulate target oncogenes, which is a key upstream regulator of the AR-V7 driven transcriptome, indicating that HoxB13 can be used as a therapeutic target for AR-V7 driven prostate tumors [147]. The discovery of HoxB13 is helpful for us to further understand how AR-V7 mediates genome function in CRPC.

Therefore, if key regulatory molecules such as AKR1C3, KIF15, DBC1 and HOXB13 can be targeted, thereby reducing the stability of AR-V7, promoting the protein ubiquitin-proteasome of AR-V7 may be able to reverse ENZR of CRPC.

## 5. Conclusions and Prospects

ENZ, a second-generation ARSI, has given hope to CRPC patients and increased the overall survival rate. Nevertheless, ENZR has become challenging in the clinical treatment of CRPC. Scientific research has also focused on the mechanism of ENZR in CRPC. Numerous studies have thus far reported that the occurrence of ENZR is mediated by different mechanisms, which are AR-related mechanisms, glucocorticoid receptor-related processes, neuroendocrine transformation, related pathway activation, etc. There is no doubt about the role of AR in mediating ENZR in CRPC. As the most important AR-Vs, AR-V7 have been shown to play an important role in the development of drug resistance in CRPC and in predicting treatment response.

In addition to expanding our understanding of the AR signal axis in the past, the research into AR-V7 in the mechanism of ENZR in CRPC also offers the most recent therapeutic approaches to treating CRPC. One of the recent research hotspots and foci are the relationships between LncRNA with proteostasis and AR-V7. In order to treat ENZR in CRPC, continuous investigation is explored on strategies targeting the stability of the AR-V7 protein or the AR-V7 transcription levels. For example, the conventional drug niclosamide has been applied in a new way. Some newly developed small-molecule compounds such as ARVib-7 and MTX-23, as well as natural products such as nobiletin and Hsp90, have also shown efficient therapeutic effects.

Although the data of preclinical studies are encouraging, the specific mechanism of AR-V7 activation is not clear at present, and most drugs are limited to the results of preclinical studies now. No further clinical trials have been carried out. In addition, there are still problems of limited efficacy or unacceptable toxicity in clinical trials in clinical development. Therefore, there is still a long way to go to develop drugs that inhibit the expression of AR-V7 or promote the degradation of AR-V7 and finally put them into clinical treatment for patients with CRPC.

## Figures and Tables

**Table 1:** Drugs and correlated mechanisms in CRPC.

Drugs	Drug Source	Clinical Development Stage of the Drug	Mechanism	Reference
C86 JG98 Uercetin VER155008	Compound medicine	Preclinical studies	Inhibit HSP40 or HSP70 and promote AR-V7 degradation.	Liu et al. (2018) [86] Kazuaki Kita et al. (2017) [98] Michael A Moses et al. (2018) [83]
KRIBB3	Compound medicine	Preclinical studies	Inhibit HSP27 and promote AR-V7 degradation	Ilker Kiliccioglu et al. (2019) [75]
Niclosamide	Compound medicine	It is an anthelminthic drug approved by FDA in 1982 and is in preclinical research in the treatment of CRPC.	Inhibition of AR-V7 transcriptional activity and down-regulation of AR-V7 protein expression.	Liu et al. (2014, 2017) [84,99] Navid Sobhani et al. (2018) [100]
ARVib	Compound medicine	Preclinical studies	Promoting AR/AR-V7 protein degradation through HSP70/STUB1.	Liu et al. (2021) [87]
Bruceantin	Compound medicine	The drug was used in phase I and phase II clinical trials for the treatment of B16 melanoma, colon 38, L1210, and P388 leukemia, but no objective tumor regression was observed, so the clinical trial was terminated. At present, the research on PCa removal is limited to preclinical research.	Inhibition of HSP90 promotes degradation of AR/AR-V7.	Sue Jin Moon et al. (2021) [88]
Nobiletin	Natural medicine	Preclinical studies	Inducing proteasomal degradation of AR-V7.	Liu et al. (2021) [92]
Huaier Extract	Natural medicine	Preclinical studies	Down-regulation of USP14 and promotes proteasomal degradation of AR/AR-V7.	Liu et al. (2021) [95]
Rut	Natural medicine	Preclinical studies	Targeting GRP78-dependent AR-V7 protein degradation.	Liao et al. (2020) [96]
MTX23	Compound medicine	Preclinical studies	Binds to the DNA binding domain of AR and VHL E3 ubiquitin ligase to promote the degradation of AR/AR-V7.	Geun Taek Lee et al. (2021) [97]
Indometacin	Compound medicine	It is a nonsteroidal anti-inflammatory drug approved by FDA in 2014 and is in preclinical research in the treatment of CRPC.	Target AKR1C3 and promote AR-V7 degradation.	Liu et al. (2019) [101] Wang et al. (2020) [102]
CX4945	Compound medicine	Preclinical studies	Target CK2 and down-regulated AR-V7 at both mRNA and protein levels.	Deng et al. (2017) [103]
Leelamine	Natural medicine	Preclinical studies	Down-regulation of AR/AR-Vs protein expression	Krishna B Singh et al. (2018) [104]
Bortezomib	Compound medicine	Approved by FDA in 2003 for the treatment of MM and MCL, its role in CRPC is in preclinical research stage.	Target NF-κB and down-regulation of AR-Vs protein expression.	R Jin et al. (2015) [105]
NaAsO2	Compound medicine	Preclinical studies	Down-regulated nuclear translocation and protein expression of AR/AR-Vs	Kim et al. (2017) [106]
ASC-J9	Compound medicine	Preclinical studies	Promote AR/AR-V7 degradation.	Shinichi Yamashita et al. (2012) [107]
Luteolin	Natural medicine	Preclinical studies	Down-regulated the expression level of AR-V7.	Aya Naiki-Ito et al. (2020) [108]
Indisulam	Compound medicine	Indisulam is an aryl sulfonamide drug with selective anticancer activity, its relationship with ENZ is in pre-clinical research stage.	Down-regulated AR-V7 mRNA expression level.	James E Melnyk et al. (2020) [109]

Abbreviations: CRPC: Castration-resistant prostate cancer; FDA: Food and drug administration; AR: Androgen receptor; AR-V: Androgen receptor splice variant; HSP: Heat shock protein; USP: ubiquitin specific peptidase; CK2: Casein kinase 2; MM: Multiple myeloma; MCL: Mantle cell lymphoma.

**Table 2:** The expression of LncRNAs in patients with CRPC affects its benefit from ENZ.

LncRNAs	LncRNAs Source	Sample Type	Number of Samples	Expression	Benefit from ENZ	Mechanism of Action	Reference
LncRNA-HOTAIR	ChIP-Seq analysis of the LNCaP PCa cells.	Cell line	/	Up	Decrease	Inhibition of AR ubiquitination.	Zhang et al. (2015) [135]
LncRNA-MALAT1	MALAT1 was initially overexpressed in non-small cell lung cancer patients.	Tissue and cell line	/	Up	Decrease	Promote AR-V7 transcription and expression.	Ren et al. (2013) [127] Chou et al. (2020) [136]
LncRNA-PRNCR1 LncRNA-PCGEM1,	Transcriptome Sequencing.	Tissue and cell line	3 human PCa and paired BPT	Up	Decrease	Promote AR transcription and activation.	Yang et al. (2013) [126]
LncRNA-PCBP1-AS1	Analysis through the data of GSE124291 and used RNA-scope to detect PCBP1-AS1 expression in a patient tissue microarray.	Tissue and cell line	BPH = 4 HSPC = 28, CRPC = 12	Up	Decrease	Enhanced deubiquitination of AR/AR-V7.	Zhang et al. (2021) [130]
LncRNA-ARLNC1	Using an integrative transcriptomic analysis of PCa cell lines and tissues (GSE56288 and GSE55064)	Tissue and cell line	/	Up	Decrease	Stabilizes AR transcripts; Induction of AR protein production.	Zhang et al. (2018) [137]
LncRNA-KDM4A- AS1	Processed microarray on LNCaP and LNCaP-AI cells and analyzed the results.	Tissue and cell line	BPH = 4 HSPC = 28 CRPC = 13	Up	Decrease	Inhibition of AR/AR -Vs degradation.	Zhang et al. (2022) [132]
LncRNA-PCAT1	Analysis through public database (TCGA) and performed RISH on CRPC and ADPC specimens.	Tissue and cell line	CRPC = 5 ADPC = 5	Up	Decrease	Activation of AKT and NF-κB signaling.	Shang et al. (2019) [123]
LncRNA-P21	Detection of LncRNA Expression in NEPC-PDX and Adenocarcinoma PDX Samples by qPCR Analysis.	Cell line	/	Up	Decrease	Inducing the neuroendocrine differentiation.	Luo et al. (2019) [133]
LncRNA-H19	Analysis of clinical NEPC queues using lncRNA sequencing pipeline, including eight queues (BCCA, WCM2, WCDT, GRID, VPC-P, VPC-M, JHMI-N and WCM1).	Tissue	/	Up	Decrease	Inducing neuroendocrine differentiation.	Neha Singh et al. (2021) [134]
LncRNA-NXTAR	Analyzed by UCSC genome browser and verification by qRT-PCR.	Tissue and cell line	35 human PCa and paired normal prostate.	Down	Increase	Inhibit expression of AR/AR-V7	Ruchi Ghildiyal et al. (2022) [131]
LncRNA-GAS5	LncRNA-GAS5 was originally found by differential cloning in growth arrested cells.	Cell line	/	Down	Increase	Inhibition of AR transcription and activation	Shidong Lv et al. (2021) [138]

Abbreviations: LncRNA: Long non-coding RNA; ENZ: Enzalutamide; AR: Androgen receptor; AR-V7: Androgen receptor splice variant 7; CRPC: Castration-resistant prostate cancer; PCA: Prostatic cancer; BPH: Benign prostatic hyperplasia; HSPC: Hormone-sensitive prostate cancer; ADPC: Androgen-dependent prostate cancer; NEPC: Neuroendocrine prostate cancer; PCR: Polymerase Chain Reaction; qPCR: Real-time quantitative PCR detection system; RISH: RNA in situ hybridization; PDX: Patient-derived tumor xenograft.

**Table 3:** Some molecules in patients with CRPC affect the therapeutic effect of ENZ.

Molecule	Therapeutic Effect of ENZ	Mechanism	Reference
KIF15	Decrease	Increase protein binding of USP14 to AR/AR-V7 to prevent degradation of AR/AR-V7 protein.	Gao et al. (2021) [142]
DBC1	Decrease	Enhance DNA binding activity of AR-V7 and inhibit ubiquitination degradation of AR-V7.	Su et al. (2017) [144]
TNC	Increase	Promote AR-V7 transcriptional activity and protein expression.	Rintu Thomas et al. (2022) [148]
STUB1	Decrease	Promote AR-V7 degradation.	Xu et al. (2020) [149] Liu et al. (2018) [86]

Abbreviations: KIF15: kinesin family member 15; DBCI: deleted in breast cancer 1; TNC: Tenascin-C; STUB1: STIP1 homology and U-box containing protein 1.

## Data Availability

The data used in this study are available from the corresponding authors.
